# Identification of Proteins Targeted by the Thioredoxin Superfamily in *Plasmodium falciparum*

**DOI:** 10.1371/journal.ppat.1000383

**Published:** 2009-04-10

**Authors:** Nicole Sturm, Esther Jortzik, Boniface M. Mailu, Sasa Koncarevic, Marcel Deponte, Karl Forchhammer, Stefan Rahlfs, Katja Becker

**Affiliations:** 1 Interdisciplinary Research Center, Justus Liebig University, Giessen, Germany; 2 Proteome Sciences R&D GmbH & Co. KG, Frankfurt am Main, Germany; 3 Institute for Physiological Chemistry, Ludwig Maximilians University, Munich, Germany; 4 Institute of Microbiology and Molecular Biology, Justus Liebig University, Giessen, Germany; Washington University School of Medicine, United States of America

## Abstract

The malarial parasite *Plasmodium falciparum* possesses a functional thioredoxin and glutathione system comprising the dithiol-containing redox proteins thioredoxin (Trx) and glutaredoxin (Grx), as well as plasmoredoxin (Plrx), which is exclusively found in *Plasmodium* species. All three proteins belong to the thioredoxin superfamily and share a conserved Cys-X-X-Cys motif at the active site. Only a few of their target proteins, which are likely to be involved in redox reactions, are currently known. The aim of the present study was to extend our knowledge of the Trx-, Grx-, and Plrx-interactome in *Plasmodium*. Based on the reaction mechanism, we generated active site mutants of Trx and Grx lacking the resolving cysteine residue. These mutants were bound to affinity columns to trap target proteins from *P. falciparum* cell extracts after formation of intermolecular disulfide bonds. Covalently linked proteins were eluted with dithiothreitol and analyzed by mass spectrometry. For Trx and Grx, we were able to isolate 17 putatively redox-regulated proteins each. Furthermore, the approach was successfully established for Plrx, leading to the identification of 21 potential target proteins. In addition to confirming known interaction partners, we captured potential target proteins involved in various processes including protein biosynthesis, energy metabolism, and signal transduction. The identification of three enzymes involved in *S*-adenosylmethionine (SAM) metabolism furthermore suggests that redox control is required to balance the metabolic fluxes of SAM between methyl-group transfer reactions and polyamine synthesis. To substantiate our data, the binding of the redoxins to *S*-adenosyl-L-homocysteine hydrolase and ornithine aminotransferase (OAT) were verified using BIAcore surface plasmon resonance. In enzymatic assays, Trx was furthermore shown to enhance the activity of OAT. Our approach led to the discovery of several putatively redox-regulated proteins, thereby contributing to our understanding of the redox interactome in malarial parasites.

## Author Summary

Protection from oxidative stress and efficient redox regulation are essential for malarial parasites which have to grow and multiply rapidly in various environments. As shown by glucose-6 phosphate dehydrogenase deficiency, a genetic variation protecting from malaria, the parasite–host cell unit is very susceptible to disturbances in redox equilibrium. This is the major reason why redox active proteins of *Plasmodium* currently belong to the most attractive antimalarial drug targets. The dithiol-containing redox proteins thioredoxin (Trx) and glutaredoxin (Grx), as well as plasmoredoxin (Plrx), which is exclusively found in *Plasmodium* species, represent central players in the redox network of malarial parasites. To extend our knowledge of interacting partners and the functions of these proteins, we carried out pull-down assays with immobilized active site mutants of Trx, Grx, and Plrx and whole cell parasite lysate. After elution of bound proteins and mass spectrometric identification, about 20 interacting partners were identified for each of the redox proteins. Data was supported using BIAcore surface plasmon resonance. The identified interacting proteins, which are likely to be redox-regulated, are involved in important cellular processes including protein biosynthesis, energy metabolism, polyamine synthesis, and signal transduction. Our results contribute to our understanding of the redox interactome in malarial parasites.

## Introduction

The malarial parasite *Plasmodium falciparum* (Pf) possesses two major functional redox systems: The thioredoxin system [1],[2] comprising NADPH, thioredoxin reductase, thioredoxin (Trx) [2],[3], and thioredoxin-dependent peroxiredoxins [4]–[8], and a glutathione system comprising NADPH, glutathione reductase [9], glutathione, glutathione *S*-transferase [10],[11], glutaredoxin (Grx) [12], and the glyoxalases I and II [13],[14].

Dithiol Trx and Grx belong to the thioredoxin superfamily whose members characteristically share the ‘thioredoxin-fold’ consisting of a central four-stranded β-sheet surrounded by α-helices [15], and an active site with two conserved cysteine residues that specify the biological activity of the protein. Besides Trx with the classical active site sequence CGPC and Grx possessing a CPYC-motif, also tryparedoxin, protein disulfide isomerase, glutathione peroxidase, glutathione *S*-transferase, and DsbA (a disulfide bond forming protein of bacteria) belong to the thioredoxin superfamily [15]–[17]. In addition, a redox-active protein named plasmoredoxin (Plrx), which is unique and highly conserved among *Plasmodium* species, has been identified and analyzed by Becker *et al.* [18]. Homology modeling of Plrx indicated a characteristic thioredoxin fold including the active site sequence WCKYC which results in assigning also Plrx to the thioredoxin superfamily [18]. The gene encoding Plrx was found to be non-essential for *Plasmodium berghei* and Plrx knock out parasites did not reveal a significant phenotype throughout the complete life-cycle [19].

The regulation of a number of phenomena in the cell has been linked to the reversible conversion of disulfides to dithiols thereby modulating the activities of the respective proteins [20]. Several recent articles have described the identification of Trx- and Grx-interacting proteins in plants and other organisms. The putative target proteins are involved in many processes, including oxidative stress response (e.g. peroxiredoxins (Prxs), ascorbate peroxidase, catalase), nitrogen, sulfur, and carbon metabolisms (e.g. *S*-adenosylmethionine synthetase, *S*-adenosyl-L-homocysteine hydrolase, phosphoglycerate kinase), protein biosynthesis (e.g. several elongation factors), and protein folding (e.g. heat shock proteins, protein disulfide isomerase) [21]–[26].

For *Plasmodium*, it has been shown that Trx as well as Grx and Plrx may operate as dithiol reductants on ribonucleotide reductase *in vitro* [1]. All three disulfide oxidoreductases are probably involved in redox regulation and/or antioxidant defense of the parasite: Trx serves as an electron donor for Plrx, oxidized glutathione disulfide and Prxs *in vitro* [1],[27],[28]. For example, the cytosolic 2-Cys peroxiredoxin of *Plasmodium* involved in the detoxification of reactive oxygen and nitrogen species and has been shown to be also fueled by plasmoredoxin [29]. Another peroxiredoxin, the so-called antioxidant protein, represents a further electron acceptor of Grx and Plrx *in vitro* [28].

The reduction of protein disulfides by Trx, Grx and Plrx is based on a dithiol exchange mechanism. The N-terminal cysteine residue of the Cys-X-X-Cys motif initiates a nucleophilic attack on the disulfide target resulting in the formation of a mixed disulfide. The intermolecular disulfide bond is subsequently cleaved by the C-terminal resolving cysteine residue of the active site motif, yielding reduced substrate and the oxidoreductase disulfide [20],[30].

Here we present redox-affinity chromatographical studies in order to gain further insight into the interactome of *Plasmodium* Trx, Grx, and Plrx by capturing potential target proteins. Our approach led to the identification of around 20 binding partners for each of the proteins applied. Furthermore, the interaction of *S*-adenosyl-L-homocysteine hydrolase (SAHH) and ornithine aminotransferase (OAT) with the redoxins was studied in more detail by surface plasmon resonance experiments.

## Results

### Identification of potential thioredoxin target proteins in *P. falciparum*

**Figure 1:**
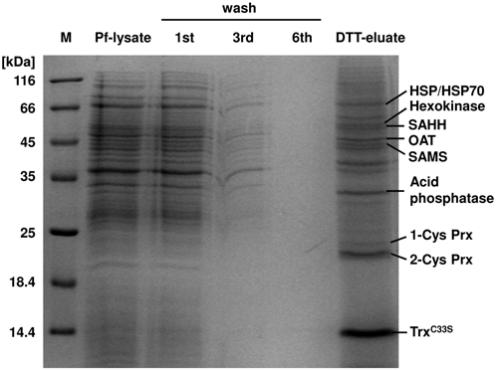
SDS-PAGE profile of the captured proteins by Trx-affinity chromatography. The thioredoxin mutant Trx^C33S^ was immobilized on CNBr-activated Sepharose 4B resin before incubating the column with 7–10 mg of *Plasmodium falciparum* cell lysate and extensive washing steps with NaCl-containing buffer. Target proteins were eluted with 10 mM DTT. The obtained protein samples were separated on a 12% polyacrylamide gel, and protein bands were identified after tryptic digestion by MALDI-TOF analysis. Some prominent proteins are indicated in the figure. For a complete list of protein bands that could be reproducibly and unambiguously assigned, please see Table 1.

Based on the dithiol exchange mechanism catalysed by Trx, we generated the active site cysteine mutant Trx^C33S^ (Table S1) which is able to catch target proteins as a mixed disulfide intermediate. This method is well established for Trx [31] and has been applied successfully to a whole range of organisms [21],[24],[32],[33]. By using the immobilized mutant as bait, we successfully captured potential target proteins from trophozoite stage *P. falciparum* cell lysate as shown in Figure 1. Elution of interacting proteins with DTT was carried out after extensive washing with NaCl-containing buffer (see wash in Figure 1) in order to remove unspecifically bound proteins and to increase specificity of the eluate fraction. (For direct comparison of the DTT eluates from Trx, Grx, and Plrx pull downs, please see Figure S1). When comparing the *P. falciparum* cell lysate fraction on the gel with the one of the eluate, it became evident that this approach facilitates an enrichment of potential target proteins on the column (e.g. see Prxs bands). The strong band for Trx^C33S^ in the eluate is likely to reflect the cleavage of dimeric bait protein (Trx^C33S^-SS-Trx^C33S^) and is not a result of inefficient washing. Captured proteins were analyzed by peptide mass fingerprinting with MALDI-MS which enabled us to identify 17 putative Trx-linked proteins that are summarized in Table 1, [34].

**Table 1:** Potential thioredoxin target proteins identified in *Plasmodium falciparum*.

Protein Name	Accession Number		Protein MW [kDa]	Peak Time Expression^a^ [Hours]	Predicted Localisation, Expression Levels^b^	Protein Isoelectric Point	Protein Coverage [%]	Masses Matched
	Swiss-Prot/GenBank	PlasmoDB						
Plasmoredoxin (Thioredoxin-like redox-active protein)	Q8I224	PFC0166w	21.7	16	c, ++	8.94	15	3
Human peroxiredoxin 2 (Prx 2)	P32119		21.8	—	c	5.67	19	4
2-Cys peroxiredoxin (2-Cys-Prx)	Q8IL80	PF14_0368	21.8	11	c, +++	6.65	44	5
1-Cys peroxiredoxin (1-Cys-Prx)	Q8IAM2	PF08_0131	25.2	25	c, +++	6.31	29	6
GTPase, putative	Q8IDL8	MAL13P1.241	26.1	36	c, +	5.55	15	3
14-3-3 protein homologue, putative	Q8IB17	MAL8P1.69	29.5	34	c, +++	4.96	23	4
Acid phosphatase, putative	Q8IM55	PF14_0036	35.8	37	c, ++	5.67	33	9
*S*-adenosylmethionine synthetase	Q7K6A4	PFI1090w	44.8	30	c, +++	6.28	21	6
Ornithine aminotransferase	Q6LFH8	PFF0435w	46.1	18	c, +++	6.47	16	5
HAP protein/Plasmepsin III	Q8IM15	PF14_0078	51.7	48	tm (api), +++	8.04	16	8
*S*-adenosyl-L-homocysteine hydrolase	Q7K6A6	PFE1050w	53.8	30	c, +++	5.64	8	3
Hexokinase	Q6LF74	PFF1155w	55.3	12	c, +++	6.72	8	3
Pyruvate kinase, putative	Q6LF06	PFF1300w	55.7	22	c, +++	7.50	17	5
Fork head domain protein, putative	Q8IEN7	PF13_0042	68.3	?	c, (++)	9.04	8	4
Heat shock protein	Q8I2X4	PFI0875w	72.4	33	sp, +++	5.18	17	8
Heat shock 70 kDa protein	Q8IB24	PF08_0054	73.9	01	c, +++	5.50	11	6
Heat shock protein 86	Q8IC05	PF07_0029	86.2	17	c, +(+)	4.94	14	7

a Data depicted from PlasmoDB and Ginsburg, Hagai. “Malaria Parasite Metabolic Pathways” (http://sites.huji.ac.il/malaria/), hours represent the 48-hour red blood cycle.

b Data depicted from PlasmoDB. If no target sequence or localization signal is predicted, the respective proteins are given as cytosolic (c).

peptide.

tm, predicted transmembrane domain; sp, predicted signal peptide; +++, expression >75%; ++, expression 50%–75%; +, expression less than 50%. Data from PlasmoDB [34].

### Identification of potential glutaredoxin target proteins in *P. falciparum*

The method described above for Trx was also used for the identification of Grx-interacting proteins. The principle of this approach has been previously established by Rouhier *et al.*, 2005, for plant Grx [25]. Grx was mutated (Grx^C32S^; Table S1) and the mutant was coupled to CNBr-activated Sepharose. We were able to identify 17 target candidates for Grx which are presented in Table 2. These proteins overlapped only partially with the proteins captured by Trx (please compare Tables 1 and 2). For example, the two glycolytic enzymes hexokinase and a putative pyruvate kinase were identified both with Grx and Trx. L-lactate dehydrogenase, however, reacted only with Grx and glyceraldehyde-3-phosphate dehydrogenase only with Grx and Plrx. Several heat shock proteins as well as enzymes involved in SAM metabolism were captured with all three bait proteins. Two ribosomal proteins involved in protein translation were found to interact exclusively with Grx or with Grx and Plrx, respectively. Interestingly, plasmoredoxin was verified as Grx- (as well as Trx-) electron acceptor which had been suggested by former *in vitro* studies [18]. A putative phosphoethanolamine N-methyltransferase was furthermore identified as a Grx-specific target.

**Table 2 ppat-1000383-t002:** Potential glutaredoxin target proteins identified in *Plasmodium falciparum*.

Protein Name	Accession Number		Protein MW [kDa]	Peak Time Expression^a^ [Hours]	Predicted Localization, Expression Levels^b^	Protein Isoelectric Point	Protein Coverage [%]	Masses Matched
	Swiss-Prot	PlasmoDB						
40S ribosomal protein S12	O97249	PFC0295C	15.4	11	c, ++	4.91	23	3
Ribosomal protein S19s, putative	Q8IFP2	PFD1055w	19.7	9	c, +++	10.17	34	6
Plasmoredoxin (Thioredoxin-like redox-active protein)	Q8I224	PFC0166w	21.7	16	c, ++	8.94	20	4
14-3-3 protein homologue, putative	Q8IB17	MAL8P1.69	29.5	34	c, +++	4.96	20	4
Phosphoethanolamine N-methyltransferase, putative	Q8IDQ9	MAL13P1.214	31.0	26	c, +++	5.43	23	4
L-lactate dehydrogenase	Q76NM3	PF13_0141	34.1	21	sp (tm), +++	7.12	28	7
Guanine nucleotide-binding protein, putative/receptor for activated C kinase homolog	Q8IBA0	PF08_0019	35.7	11	c, +++	6.24	25	4
Glyceraldehyde-3-phosphate dehydrogenase	Q8IKK7	PF14_0598	36.6	12	c, +++	7.59	21	5
*S*-adenosylmethionine synthetase	Q7K6A4	PFI1090w	44.8	30	c, +++	6.28	9	3
Ornithine aminotransferase	Q6LFH8	PFF0435w	46.1	18	c, +++	6.47	14	6
*S*-adenosyl-L-homocysteine hydrolase	Q7K6A6	PFE1050w	53.8	30	c, +++	5.64	14	4
Hexokinase	Q6LF74	PFF1155w	55.3	12	c, +++	6.72	11	4
Pyruvate kinase, putative	Q6LF06	PFF1300w	55.7	22	c, +++	7.50	36	12
Heat shock protein	Q8I2X4	PFI0875w	72.4	33	sp, +++	5.18	11	6
Heat shock 70 kDa protein	Q8IB24	PF08_0054	73.9	01	c, +++	5.50	17	8
Heat shock protein 86	Q8IC05	PF07_0029	86.2	17	c, +(+)	4.94	20	14
Elongation factor 2	Q8IKW5	PF14_0486	93.5	13	c, +++	6.35	17	11

a Data depicted from PlasmoDB and Ginsburg, Hagai. “Malaria Parasite Metabolic Pathways” (http://sites.huji.ac.il/malaria/), hours represent the 48-hour red blood cycle.

b Data depicted from PlasmoDB. If no target sequence or localization signal is predicted, the respective proteins are given as cytosolic (c).

tm, predicted transmembrane domain; sp, predicted signal peptide; +++, expression >75%; ++, expression 50%–75%; +, expression less than 50%. Data from PlasmoDB [34].

### Identification of potential plasmoredoxin target proteins in *P. falciparum*

Plrx was first described in 2003 by Becker *et al.* [18] and is unique for and highly conserved among *Plasmodium* species. So far, the physiological function of Plrx is not completely understood, and the protein is not essential for the survival of the parasite as shown very recently by Buchholz and co-workers [19]. To gain further insight, we generated an active site mutant (Plrx^C63S^; Table S1) in analogy to Trx and Grx. The mutant was then used for affinity chromatography resulting in 21 proteins which are potential electron acceptors of Plrx (Table 3). Among several overlapping proteins also captured with Trx or Grx, Plrx was shown to specifically interact with the putative co-chaperone GrpE and a putative disulfide isomerase, both assisting in protein folding, as well as with a putative acyl carrier protein and enzymes involved in DNA synthesis, -repair, and signal transduction.

**Table 3:** Potential plasmoredoxin target proteins identified in *Plasmodium falciparum*.

Protein Name	Accession Number		Protein MW [kDa]	Peak Time Expression^a^ [Hours]	Predicted Localization, Expression Levels^b^	Protein Isoelectric Point	Protein Coverage [%]	Masses Matched
	Swiss-Prot/ GenBank	PlasmoDB						
40S ribosomal protein S12	O97249	PFC0295C	15.4	11	c, ++	4.91	23	3
Acyl carrier protein, putative	Q7KWJ1	PFB0385w	15.8	23	sp (api), ++	8.87	19	3
14-3-3 protein homologue, putative	Q8IB17	MAL8P1.69	29.5	34	c, +++	4.96	20	4
Co-chaperone GrpE, putative	Q8IIB6	PF11_0258	34.5	19	n, ++	8.8	20	4
14-3-3 Protein, putative	Q8ID86	MAL13P1.309	35.1	01	tm, +	7.08	17	3
Glyceraldehyde-3-phosphate dehydrogenase	Q8IKK7	PF14_0598	36.6	12	c, +++	7.59	30	7
Hypothetical protein	Q8IJX6	PF10_0065	37.8		c, +	8.67	16	4
Ribonucleotide reductase small subunit (R2)	Q8IM38	PF14_0053	40.6	33	c, ++	5.37	14	4
*S*-adenosylmethionine synthetase	Q7K6A4	PFI1090w	44.8	30	c, +++	6.28	20	5
Conserved GTP-binding protein, putative	Q8IBM9	MAL7P1.122	45.2	11	c, ++	6.88	35	9
Ornithine aminotransferase	Q6LFH8	PFF0435w	46.1	18	c, +++	6.47	22	8
Endonuclease iii homologue, putative	Q6LFC2	PFF0715c	49.2	26	c, +	9.37	11	4
HAP protein/Plasmepsin III	Q8IM15	PF14_0078	51.7	48	tm (api), +++	8.04	18	8
Hypothetical protein	Q8ILQ4	PF14_0190	54.7		c, ++	5.53	9	3
Hexokinase	Q6LF74	PFF1155w	55.3	12	c, +++	6.72	11	4
Disulfide isomerase, putative	Q8I6S6	MAL8P1.17	55.5	31	sp (tm),++	5.56	9	3
Pyruvate kinase, putative	Q6LF06	PFF1300w	55.7	22	c, +++	7.5	28	9
Heat shock protein	Q8I2X4	PFI0875w	72.4	33	sp, +++	5.18	9	6
Heat shock 70 kDa protein	Q8IB24	PF08_0054	73.9	01	c, +++	5.5	28	12
Heat shock protein 86	Q8IC05	PF07_0029	86.2	17	c, +(+)	4.94	13	8
Elongation factor 2	Q8IKW5	PF14_0486	93.5	13	c, +++	6.35	10	5

a Data depicted from PlasmoDB and Ginsburg, Hagai. “Malaria Parasite Metabolic Pathways” http://sites.huji.ac.il/malaria/, hours represent the 48-hour red blood cycle.

b Data depicted from PlasmoDB. If no target sequence or localization signal is predicted, the respective proteins are given as cytosolic (c).

tm, predicted transmembrane domain; sp, predicted signal peptide; n, predicted nuclear localization with mitochondrial signal sequence, +++, expression >75%; ++, expression 50%–75%; +, expression less than 50%. Data from PlasmoDB [34].

### BIAcore interaction analysis of *S*-adenosyl-L-homocysteine hydrolase with Trx, Grx, and Plrx

To further investigate the interaction between the redoxins and newly captured proteins we cloned, overexpressed and purified *S*-adenosyl-L-homocysteine hydrolase and ornithine aminotransferase. These two proteins were chosen since they both are involved in SAM metabolism and they could be produced without major problems in recombinant form. In a first step the interaction of SAHH, which was trapped with the Trx and Grx affinity column, and OAT (see below) with the three redox-active proteins was investigated by BIAcore surface plasmon resonance (SPR) analyses. Oxidized SAHH was attached covalently to the sensor chip surface, and various forms of the redox-active proteins, including wild type and active site mutants lacking one or two cysteines, were used as analytes.

**Figure 2:**
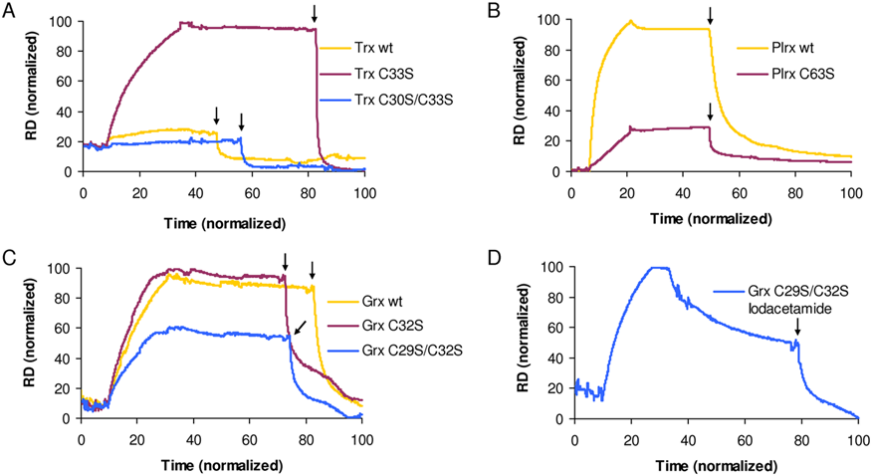
SPR analysis of S-adenosyl-L-homocysteine hydrolase (SAHH) with Trx, Grx, and Plrx. SAHH on the sensor surface was oxidized with 30 µl of 0.5 mM 5,5′-dithiobis(2-nitrobenzoate) (DTNB) (not shown). Then, 30 µl of the analyte (10 µM in HBS buffer) was injected, followed by buffer flow over the chip. The dissociation phase was initiated by addition of 30 µl of a 2 mM DTT solution as indicated by an arrow. The BIAcore sensorgrams show the response difference (RD) between SAHH-loaded flow chamber 2 and control flow chamber 1 (FC2–FC1). (A–C) Interaction of SAHH with wild type and active site mutants of Trx, Grx, and Plrx. In order to analyze a non-covalent interaction of Grx with SAHH, the Grx^C29S/C32S^ mutant was treated with iodoacetamide prior to SPR analysis (D).

Figure 2A shows the sensorgram of Trx binding to SAHH. Trx^C33S^, the mutant used in the pull-down assay, associated strongly with SAHH, and the binding was not affected during buffer flow over the chip which is indicative for a covalent interaction. The fact that the protein complex could efficiently be dissociated by DTT confirms the existence of a disulfide bond between the two proteins. In contrast, wild type Trx as well as Trx^C30S/C33S^ which lack both active site cysteines caused no obvious increase in resonance units.

For Plrx, the interaction of the wild type protein with SAHH was much stronger as for the single cysteine mutant. Both forms, however, bound stable to SAHH and dissociated upon addition of DTT (Figure 2B).

The sensorgram in Figure 2C shows the interaction of SAHH with Grx, which was comparable for the wild type protein and the cysteine mutant Grx^C32S^. Interestingly, also the double mutant of Grx was able to form a covalent complex with SAHH, the interaction, however, was not as intense as for the two other variants. Grx possesses, apart from the two active site cysteines, one additional cysteine in its sequence (Cys 88). In order to verify if the observed interaction is due to a disulfide bridge between residue Cys 88 and SAHH, the double mutant was treated with iodoacetamide resulting in an alkylation of the thiol group. Indeed, this Grx form with a modified Cys 88 reacted with SAHH in a mainly non-covalent manner as shown in Figure 2D. Only a part of the Grx could be eluted with DTT which is presumably due to incomplete alkylation.

### Interaction between ornithine amino transferase and thioredoxin

**Figure 3:**
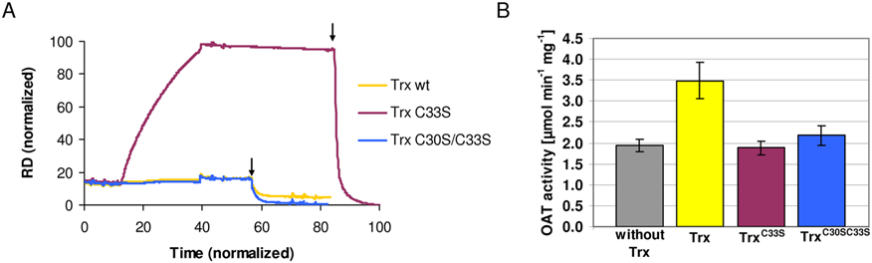
SPR analysis and specific activity of ornithine aminotransferase (OAT) in the presence of Trx. (A) SPR analysis of OAT with Trx. The experimental setup was chosen as described in the Figure 2 caption. A strong and specific interaction can be observed with the C33S mutant, whereas wild type Trx and the double mutant C30S/C33S show hardly any interaction. (B) Specific activity of PfOAT in the presence of equimolar concentrations of Trx, TrxC33S, and TrxC30S/C33S.

As a second protein captured by the redoxins in the pull down assays, we studied ornithine aminotransferase (OAT) in more detail. Here we focused on SPR and enzyme kinetic analyses with Trx. As described above for the SAHH SPR experiments, OAT was attached covalently to the sensor chip surface and wild type Trx as well as active site single and double mutants, lacking one or two cysteines, were used as analytes. Figure 3A shows the sensorgram of Trx binding to OAT. Trx^C33S^, the mutant used in the pull-down assay, associated strongly with OAT. This interaction could not be disturbed by washing but could be specifically solved by DTT indicating a disulfide bond formation between OAT and the Trx mutant. Again, as observed for SAHH, wild type Trx as well as Trx^C30S/C33S^ which lacks both active site cysteines showed no obvious interaction.

The biological significance of the interaction between Trx and OAT was further studied in enzymatic assays (Figure 3B). Interestingly, the addition of equimolar concentrations of wild type thioredoxin to the assay resulted in 75% increase in OAT activity. In contrast, the Trx single and double mutants did not induce activity changes which indicates that an intact active site with two cysteine residues is required for the activation of OAT.

## Discussion

In the present study, we intended to identify novel target proteins of *P. falciparum* Trx, Grx, and Plrx in order to extend our knowledge of their physiological functions. An affinity chromatography approach using active site cysteine mutants as bait had already been described for Trx [21], [24], [31]–[33] and Grx [25] from other organisms. Here, we applied this strategy successfully to *P. falciparum* Trx and Grx as well as to another member of the thioredoxin superfamily, namely Plrx. For Trx and Grx, 17 (partially overlapping) target candidates were detected each (Tables 1 and 2). Most of these proteins had not previously been associated with redox-mediated regulation in *P. falciparum*. Furthermore, our approach represents the first interactome study carried out with a plasmoredoxin. For Plrx we identified 21 putative target proteins (Table 3). Taken together 33 different putative interacting proteins were identified with these three redoxins, a number which fits to results obtained on other organisms with Trx [31] (Table 1). All target proteins identified are present in asexual red blood cell stages of the parasites (PlasmoDB, see Tables 1–3) with most proteins predicted to be cytosolic. The same cytosolic localization is predicted for our three bait redoxins.

The different target proteins found are involved in various metabolic processes that can be clustered as summarized in Table 4. The physiological relevance of the observed interactions between the target proteins and our redox-active proteins will be studied in further detail. For *S*-adenosyl-L-homocysteine hydrolase, a central enzyme of *S*-adenosylmethionine metabolism, and ornithine aminotransferase the interaction was verified exemplarily and studied in more detail using BIAcore surface plasmon resonance (SPR) analyses. For OAT also activity assays were carried out to show the influence of thioredoxin on the physiological reaction of the enzyme (see below).

**Table 4:** Functional clusters of PfTrx1, PfGrx1, and PfPlrx target protein candidates captured in the present study.

**Anti-Oxidative Stress System**			
Trx			2-Cys peroxiredoxin (2-Cys-Prx, cytosolic)
Trx			1-Cys peroxiredoxin (1-Cys-Prx, cytosolic)
Trx			Human peroxiredoxin 2
Trx	Grx		Plasmoredoxin (Thioredoxin-like redox-active protein)
**Transcription/Translation**			
Trx			Fork head domain protein, putative
	Grx	Plrx	Elongation factor 2
	Grx	Plrx	40S ribosomal protein S12
	Grx		Ribosomal protein S19s, putative
**Protein Folding**			
Trx	Grx	Plrx	Heat shock protein
Trx	Grx	Plrx	Heat shock 70 kDa protein
Trx	Grx	Plrx	Heat shock protein 86
		Plrx	Co-chaperone GrpE, putative
		Plrx	Disulfide isomerase, putative
**Carbohydrate Metabolism**			
Trx	Grx	Plrx	Hexokinase
Trx	Grx	Plrx	Pyruvate kinase, putative
	Grx	Plrx	Glyceraldehyde-3-phosphate dehydrogenase
	Grx		L-lactate dehydrogenase
**SAM Metabolism**			
Trx	Grx	Plrx	Ornithine aminotransferase
Trx	Grx	Plrx	S-adenosylmethionine synthetase
Trx	Grx		S-adenosyl-L-homocysteine hydrolase
**Lipid Metabolism**			
	Grx		Phosphoethanolamine N-methyltransferase, putative
		Plrx	Acyl carrier protein, putative
**Hemoglobin Catabolism**			
Trx		Plrx	HAP protein/Plasmepsin III
**DNA Synthesis and Repair**			
		Plrx	Endonuclease iii homologue, putative
		Plrx	Ribonucleotide reductase small subunit
**Signal Transduction**			
Trx	Grx	Plrx	14-3-3 protein homologue, putative
		Plrx	14-3-3 protein, putative
Trx			GTPase, putative
	Grx		Guanine nucleotide-binding protein, putative/receptor for activated C kinase homolog, PfRACK
		Plrx	Conserved GTP-binding protein, putative
**Others**			
Trx			Acid phosphatase, putative
		Plrx	Hypothetical protein
		Plrx	Hypothetical protein

### Antioxidant enzymes and redox control

Peroxiredoxins, repeatedly identified as target proteins in our study, represent central antioxidant and redox-regulatory proteins in *Plasmodium*. This notion is underlined by the fact that malarial parasites possess neither catalase nor a classical glutathione peroxidase [1]. Many Prxs are present at high intracellular concentrations and are therefore detected in almost every proteomic analysis. From the four *P. falciparum* Prxs both cytosolic proteins, 2-Cys-Prx and 1-Cys-Prx, were identified in our study using Trx as a bait. On the other hand, no peroxiredoxin was captured with Grx or Plrx (Tables 2 and 3). Since cytosolic 2-Cys-Prx and 1-Cys-Prx are both highly abundant redox proteins, the results suggest an excellent specificity of our method (e.g. with Grx and Plrx as negative controls for potential interacting partners of Trx). Our data support former *in vitro* studies indicating that Trx is the preferred physiological electron donor of these *Plasmodium* peroxiredoxins having distinct antioxidant and regulatory functions i*n vivo* [1],[28],[35],[36]. The other two Prxs from *P. falciparum* as well as a glutathione peroxidase-like enzyme, were not found in our study. This might be due to the lower protein abundance or due to the subcellular localization resulting in different substrate specificities (although the three enzymes were shown to accept electrons from at least one of the bait proteins *in vitro*) [28], [35]–[37]. Detection of an erythrocytic Prx in the pull-down with Trx (Table 1) might indicate a contamination during lysate preparation. However, very recently we showed that *P. falciparum* can import the human peroxiredoxin 2 into its cytosol, suggesting that the parasite exploits this antioxidant system of its host (Koncarevic *et al.*, under revision). Non-recombinant plasmoredoxin was captured from parasite lysates with Trx and Grx (Tables 1 and 2). Also this interaction had been described before by biochemical assays and was now verified [18]. It is noteworthy that no other member of the thioredoxin superfamily was found as a target of plasmoredoxin although *P. falciparum* has many Trx- and Grx-like proteins [1],[28]. One might therefore speculate that Plrx is involved in the cross talk of the thioredoxin and the glutaredoxin system.

### Protein biosynthesis

Based on our results, some components of the translational machinery seem to be redox-regulated in malarial parasites. Two ribosomal proteins and the elongation factor 2 were identified as redox sensitive targets in *Plasmodium* (Table 4) and had also been described as Trx or Grx targets in other organisms [21]–[23],[25],[26],[32]. The redox sensitivity of elongation factor 2 has been confirmed *in vitro* and *in vivo*. Analyses of the effect of oxidative stress on protein synthesis indicated that elongation factor 2, the main protein involved in the elongation step, is oxidatively modified resulting in lower amounts of active protein [38]. Besides, several redox-dependent chaperones were identified in our work (Table 4). Hsp70 had already been linked to Trx and Grx in plants, *Chlamydomonas* and *Synechocystis* [21]–[23],[25],[26],[33]. A recent study demonstrated the formation of a complex between an *Arabidopsis* Trx-like protein and yeast Hsp70 that is released under oxidative stress. Cysteine 20 which is conserved in virtually all the Hsp70 chaperones has been suggested as target of redox regulation [39]. The existence of a redox-regulated molecular chaperone network has been described by Hoffmann and coworkers focusing on Hsp33 which is reduced *in vivo* by the glutaredoxin and thioredoxin systems [40]. The present work adds the *Plasmodium* protein disulfide isomerase to the list of known redox-dependent components involved in protein folding [22],[25],[26]. Thioredoxin was reported to regulate translation via an interaction with a protein disulfide isomerase, namely RB60, in the green algae *Chlamydomonas* [41].

### Carbohydrate metabolism

Glyceraldehyde-3-phosphate dehydrogenase (GAPDH), which was found to interact with Grx and Plrx in our study, had been described previously as a protein undergoing thiol/disulfide redox status changes which affect its enzymatic activity. Molecular modeling studies of plant GAPDH indicated a disulfide bond between an N-terminal and C-terminal cysteine being involved in redox regulation, which alters the geometry of the active site [42],[43]. Two of eight cysteines of PfGAPDH are strictly conserved among prokaryotes and eukaryotes: Cys 153 which is directly involved in catalysis, and the near-neighbor Cys 157 which is located on the same helical segment at a distance of 8.6 Å (PDB entry 2B4T; [44]). Indeed, Cys 149 of rabbit muscle GAPDH has been identified as target for the oxidation by peroxynitrite [45]. A reversible disulfide formation of GAPDH under oxidizing conditions was furthermore observed in vertebrate cells [46],[47], *S*-glutathionylation, however, did not inactivate the enzyme [48]. Several chloroplast enzymes including GAPDH possess the unique property of being activated by reduced thioredoxins in the light [49],[50]. In a number of former studies on plants, GAPDH had also been identified to be redox-regulated by members of the thioredoxin superfamily [25],[26],[33].

In addition to GAPDH, two other glycolytic enzymes, namely hexokinase and pyruvate kinase, as well as L-lactate dehydrogenase were captured in our study. Most of the enzymes involved in glycolysis are sulfhydryl proteins sensitive to oxidation suggesting that they may be controlled by the redox state of the cells [51],[52]. Possibly, the *Plasmodium* enzymes are regulated by similar mechanisms involving redox-active proteins. A putative redox interaction, however, remains to be studied with recombinant enzymes in detail. In this context, one should mention that the glycolytic enzyme enolase seems to be redox-regulated by a cytosolic thioredoxin system in a limited number of plant species [53].

### Hemoglobin catabolism

Hemoglobin is a major nutrient source during the intraerythrocytic life stage of *P. falciparum* and is initially processed by four different but homologous proteases, namely plasmepsins I, II, and IV and histo-aspartic protease (HAP; plasmepsin III), located in the acidic food vacuole of the parasite. Plasmepsins possess active site aspartates one of which is substituted in the HAP enzyme by histidine [54]. Among all *Plasmodium* species, four plasmepsin cysteines are strictly conserved and form two disulfide bonds which are located on the surface of the proteins (PDB entries 2ANL [55], 1MIQ [56], 1LEE [57]). These disulfides are potentially accessible for an interaction with redox-active proteins. Our results indicate an interaction of Trx and Plrx with plasmepsin III. The presence of these two redox-active proteins and their respective reducing backup systems in the food vacuole for regulation of enzyme activities seems to be very unlikely. One might, however, speculate that the HAP protein is putatively redox-regulated by cytosolic dithiol proteins on the way to its destination, the food vacuole. Of course, this hypothesis needs to be substantiated by further detailed studies.

### Signal transduction

Increasing attention has been paid to the roles of thioredoxin as a key molecule in redox signaling beyond its intrinsic antioxidant activity. For *Plasmodium*, an involvement of redox-active proteins in signal transduction has not been elucidated so far. In our study, we detected a GTPase and two guanine nucleotide-binding proteins (Table 4) which had not been described in the context of redox control before. In addition, two 14-3-3 proteins were captured with our redox-active proteins (Table 4) which confirm the findings of Rouhier *et al.* [25] who first demonstrated the redox-regulation of a 14-3-3 protein in plants. Vice versa, a global proteomic analysis aimed at identifying proteins that bind to 14-3-3 proteins during interphase and mitosis revealed Trx and a peroxiredoxin as binding partners [58]. 14-3-3 is an evolutionarily conserved protein that is most noted as a mediator in signal transduction events and cell cycle regulation [59]. In a very recent investigation conducted by Aachmann *et al.* [60], the interaction of selenoprotein W with 14-3-3 proteins had been studied using NMR. Selenoprotein W has a thioredoxin-like fold with a CXXU motif located in an exposed loop similar to the redox-active site in thioredoxin. The specific interaction which is suggested to be physiologically relevant involves the active site residues CXXU and indicates that 14-3-3 are redox-regulated proteins. These findings support the possible role of a redox-based signaling network in *Plasmodium* that needs to be further unraveled.

### *S*-adenosylmethionine (SAM) metabolism and interaction analysis of SAHH with redox-active proteins

**Figure 4:**
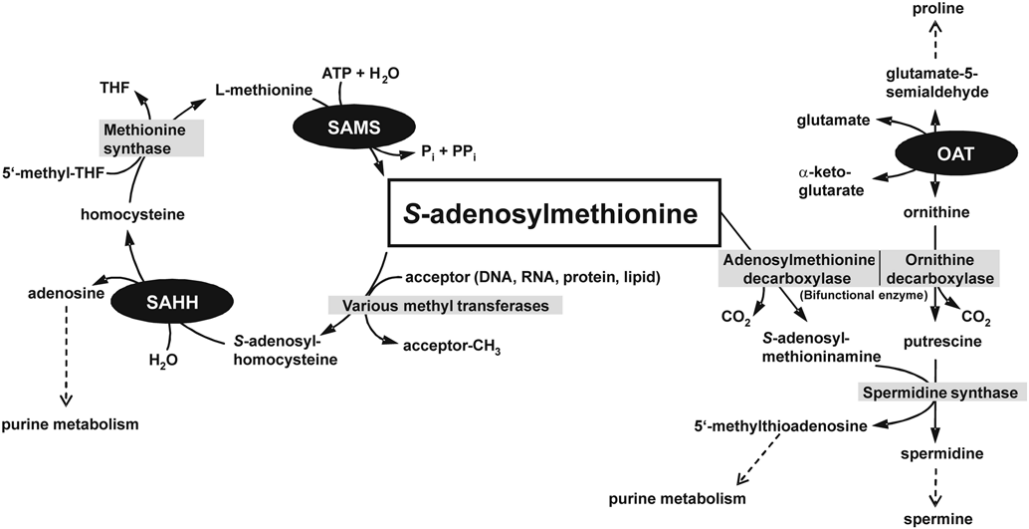
Overview of the *S*-adenosylmethionine metabolism in *P. falciparum*. On the left side, the C1 metabolism is shown comprising the putatively redox-regulated proteins SAMS and SAHH identified in the present work. OAT, which is also suggested as being redox-sensitive, is involved in the formation of ornithine, an important precursor for polyamine synthesis, which is shown on the right side. The trans-sulfuration pathway from homocysteine to cysteine has not been included in the figure. See text for details.

Three enzymes involved in *S*-adenosylmethionine metabolism, ornithine aminotransferase (OAT), *S*-adenosylmethionine synthetase (SAMS), and *S*-adenosyl-L-homocysteine hydrolase (SAHH) have been identified in this study to interact with Trx, Grx and Plrx. The first function of activated methionine in SAM is to serve as a methyl-group donor (Figure 4, left side): SAM is formed from methionine and ATP by SAMS. SAM-dependent methylation reactions lead to formation of *S*-adenosyl-L-homocysteine which is hydrolyzed into L-homocysteine and adenosine by SAHH. A putative regulation of SAMS by Trx [22],[26], as well as SAHH by Grx had been described before [25]. Methionine synthase catalyzes the reaction step between SAHH and SAMS in the “activated methyl cycle” and also represents an established target for Trx and Grx [25],[26]. Thus it is very likely that regeneration of the methyl-group donor SAM is tightly controlled by redox regulation in *P. falciparum* and other organisms. OAT which is required for the formation of ornithine has not been described in the context of redox control. However, such a regulation is quite logical considering another function of SAM. The second function of activated methionine in SAM is to serve (after decarboxylation) as the aminoalkyl-donor for the synthesis of polyamines (Figure 4, right side). This is also the case for ornithine which reacts (after decarboxylation) with the SAM-derivative to form spermidine. Thus, we hypothesize that redox regulation of OAT and SAMS is coupled to the tight control of polyamine synthesis. We furthermore suggest that regulation of OAT and the other enzymes is required to balance the metabolic fluxes of SAM between methyl-group transfer reactions and polyamine synthesis (Figure 4).

SPR analyses were carried out in order to identify the cysteine residue(s) of the three redox-active proteins on which the interaction with oxidized SAHH is based (Figure 2). For Trx, only the Trx^C33S^ mutant was able to bind to SAHH suggesting that a disulfide bond between SAHH and residue Cys 30 at the Trx active site is responsible for the interaction (Figure 2A). The double mutant could probably not be captured due to the lack of a reactive cysteine residue. For wild type Trx strikingly different redox potentials of SAHH and Trx might favor a complete reaction resulting in reduced SAHH and oxidized Trx without trapping the short-lived intermolecular disulfide intermediate. The fact that the intermediate during the reaction of oxidized SAHH with wild type Plrx (Figure 2B) is more pronounced or stable than for wild type Trx might be explained by a more positive redox potential of Plrx. The reduced amount of intermediate seen for Plrx^C63S^ might be due to a significantly altered redox potential of the mutant. The reduced stability of the intermediate might be also the reason why SAHH was not captured with the Plrx affinity column. Besides the redox interaction of the Grx active site cysteine residue(s) with SAHH, Grx is capable of forming a disulfide bond with SAHH via Cys 88 (Figure 2C). The residue is quite conserved (see alignment in ref. [12]) and is located at the active site between the GlyGly-turn ending β-strand four and α-helix four. The homologous residue Cys 117 in yeast Grx5 was also shown to be involved in redox reactions *in vitro* [61]. Which of the Grx cysteine residues acts mainly as electron donor under physiological conditions remains speculative, although the significance of the N-terminal active site cysteine for disulfide formation was substantiated by the study of Rouhier *et al.* [25]. Furthermore, the non-covalent interaction observed for Grx (Figure 2D) supports the presence of a specific protein-protein interaction between Grx and SAHH.

In addition to SAHH, SPR experiments were carried out for ornithine amino transferase, another protein captured by the pull down assays, and thioredoxin. Figure 3A shows a strong interaction of OAT with the active site single mutant of Trx. This interaction was based on a disulfide bond formation as proven by the fact that it could be solved rapidly and efficiently by DTT. In contrast to the single mutant, the Trx active site double mutant and the wild type Trx showed hardly any interaction with OAT. The biological significance of these data was studied by activity assays. As indicated in Figure 3B, the addition of equimolar amounts of wild type thioredoxin to the assay resulted in 75% increase in OAT activity. As expected, the Trx single and double mutants did not induce activity changes which indicates that an intact active site motif with two cysteine residues is required for the activation of OAT. Our data substantiate the above hypothesis that in *Plasmodium falciparum* the activity of OAT is redox-regulated. To our knowledge this is the first time that such a regulation has been proposed for ornithine aminotransferase.

### Further putative redox-linked processes in *Plasmodium*

Class I ribonucleotide reductase has been suggested to be a substrate for Plrx according to *in vitro* assays [18] and was now also identified as a Plrx interacting partner by affinity chromatography (Table 3). It should be noted that the small subunit (R2) of ribonucleotide reductase identified in our study is not the electron acceptor oxidizing dithiol proteins but generates the radical required for catalysis [62]. Either the interaction between R2 and Plrx was indirect and subunit R1 was overlooked in the mass spectrometric analyses or Plrx interacted directly with one of the eight cysteine residues of R2. The latter possibility is quite speculative but might point to a novel regulatory function of R2. Identification of a phosphoethanolamine N-methyltransferase and the acyl carrier protein suggests a potential redox regulation of lipid metabolism in malarial parasites. Furthermore, a putative redox sensitivity of an endonuclease iii homologue involved in DNA repair has, according to our knowledge, not been described before. The same holds true for an acid phosphatase whose function in *Plasmodium* remains to be studied in detail.

In order to determine if the interaction of the captured proteins with the members of the thioredoxin family and the resulting putative redox changes are of biological relevance - as started for OAT - further biochemical, biophysical and cell-biological studies will have to be conducted in the next years. Among others, these studies will include cloning, expression, mutagenesis and purification of the interacting proteins, assessment of protein-protein interactions under quasi-physiological conditions, enzyme kinetic studies in different redox environments, modeling, cocrystallization, and x-ray crystallographic or NMR analyses of protein-redoxin complexes, and knock out/knock down experiments. The expected data will further enhance our knowledge on redox regulatory processes in *Plasmodium*, on host-parasite interactions and on the potential of redox metabolism as antimalarial drug target.

## Materials and Methods

All chemicals used were of the highest purity available and were obtained from Roth (Karlsruhe, Germany), Sigma-Aldrich (Steinheim, Germany), or Merck (Darmstadt, Germany). PCR primers were obtained from MWG-Biotech (Ebersberg, Germany). The vectors pHSG398, pET28a and pDrive were obtained from Takara (Japan), Novagen (Darmstadt, Germany) and Qiagen (Hilden, Germany), respectively. The expression system QIAexpress, comprising vector pQE30, *E. coli* host strain M15, and Ni-NTA agarose resin, was purchased from Qiagen (Hilden, Germany) and *E. coli* strain BL 21 from Novagen (Darmstadt, Germany). CNBr-activated Sepharose 4B was obtained from GE Healthcare (München, Germany). RPMI 1640 medium was from Gibco (Paisley, UK).

### Site-directed mutagenesis of *PfTrx1* (PF14_0545), *PfGrx1* (PFC0271c), and *Plrx* (PFC0166w), heterologous expression and protein purification

Cloning of *PfTrx1*, *PfGrx1*, and *Plrx* has been described previously [3],[12],[18]. Mutations of *PfTrx1^C33S^*, *PfTrx1^C30S/C33S^*, *PfGrx1^C32S^*, *PfGrx1^C29S/C32S^*, and *Plrx^C63S^* were introduced by PCR with *Pfu* polymerase (Promega Corp.) using mutated primers (Table S1). Methylated non-mutated template plasmids were digested with *Dpn*I, and competent XL1-Blue cells were subsequently transformed. The introduction of the correct mutation was confirmed by sequencing.

pQE30 constructs of wild type and mutant genes were expressed in *E. coli* strain M15 (Qiagen). Cells containing the respective plasmid were grown at 37°C in LB medium supplemented with carbenicillin (100 µg/ml) and kanamycin (50 µg/ml) to an optical density at 600 nm of 0.5 to 0.6, and expression was subsequently induced for 4 h with 1 mM isopropyl-β-D-1-thiogalactopyranoside. Cells were harvested, resuspended in 50 mM sodium phosphate, 300 mM NaCl, pH 8.0, and sonicated in the presence of protease inhibitors. After centrifugation, the supernatant was applied to a Ni-NTA column, and recombinant proteins were eluted with buffer containing 75 mM imidazole. For coupling to a CNBr-activated Sepharose 4B, the respective proteins were dialysed against 100 mM NaHCO_3_, 500 mM NaCl, pH 8.3.

### Cultivation of *Plasmodium falciparum* and preparation of parasite cell extract

Intraerythrocytic stages of *P. falciparum* were grown in continuous culture as described by Trager and Jensen [63], with slight modifications. Parasites were maintained at 1 to 10% parasitemia and 3.3% hematocrit in an RPMI 1640 culture medium supplemented with A+ erythrocytes, 0.5% lipid-rich bovine serum albumin (Albumax), 9 mM (0.16%) glucose, 0.2 mM hypoxanthine, 2.1 mM L-glutamine, and 22 µg/ml gentamicin. All incubations were carried out at 37°C in 3% O_2_, 3% CO_2_, and 94% N_2_. Synchronization of parasites in culture to ring stages was carried out by treatment with 5% (w/v) sorbitol [64]. Trophozoite stage parasites were harvested by suspending the red cells for 10 minutes at 37°C in a 20-fold volume of saponin lysis buffer containing 7 mM K_2_HPO_4_, 1 mM NaH_2_PO_4_, 11 mM NaHCO_3_, 58 mM KCl, 56 mM NaCl, 1 mM MgCl_2_, 14 mM glucose, and 0.02% saponin, pH 7.4. The saponin lysis was repeated twice before washing the parasites with PBS and freezing the parasite pellet at −80°C.

For preparing the parasite cell extract, the pellet was diluted in an equal volume of buffer containing 100 mM Tris, 500 mM NaCl, pH 8.0. Parasites were disrupted by four cycles of freezing in liquid nitrogen and thawing in a waterbath at room temparature followed by sonication at 4°C. After centrifugation at 100,000 g for 30 min at 4°C, the obtained supernatant was used as cell lysate for affinity chromatography columns.

### Capturing of the target proteins by immobilized mutants of PfTrx1, PfGrx1, and Plrx

1 mg of the respective pure mutants PfTrx1^C33S^, PfGrx1^C32S^, and Plrx^C63S^ in coupling buffer (100 mM sodium carbonate, 500 mM NaCl, pH 8.3) was incubated for 1 h at room temperature under gentle agitation with 10 µl CNBr-activated Sepharose 4B resin, which had been swelled in 1 mM HCl according to the manufacturer's instructions. After termination of the coupling reaction by centrifugation and washing of the resin with coupling buffer, unmodified reactive groups of the resin were blocked by incubation with 100 mM Tris, pH 8.0 for 2 h at room temperature. *Plasmodium falciparum* cell lysate (∼800 µl) containing 7–10 mg protein was incubated with 10 µl of the liganded resin at room temperature for at least 2 h under gentle stirring, before washing the resin with 100 mM Tris, 500 mM NaCl, pH 8.0 to remove non-specifically bound proteins. The washing steps were repeated until the absorbance of the washing solution at 280 nm became zero. Finally, 10 µl resin were suspended in 22 µl 100 mM Tris, 500 mM NaCl, 10 mM DTT, pH 8.0, and were incubated for 30 min at room temperature. Elution was repeated with 12 µl elution buffer, and eluates were pooled. The eluted proteins were separated by SDS polyacrylamide gel electrophoresis. Bands of interest were excised, and then subjected to tryptic digestion and MALDI-TOF analysis as described below. To verify that the interaction between the redoxins and the captured proteins was specific and based on the proposed disulfide-dependent mechanism a number of control pull down experiments was performed. As bait proteins wild type Trx, Grx and Plrx as well as the double mutants of Trx (C30S/C33S) and Grx (C29S/C32S) were employed. In addition, CNBr-activated Sepharose 4B resin without immobilized protein was used to study unspecific binding of proteins. These control experiments were carried out in analogy to the original pull downs. As shown in Figure S2, hardly any bands could be detected in these controls indicating that the binding to the single mutants of Trx, Grx and Plrx in our pull down experiments was specific.

### Protein identification by peptide mass fingerprinting with MALDI-MS

Excised gel pieces were destained with 25 mM NH_4_HCO_3_ in 50% acetonitrile by washing them three times for 10 min each. The gel pieces were vacuum-dried and then incubated with modified porcine trypsin (Promega) at a final concentration of 0.1 mg/ml in 25 mM NH_4_HCO_3_, pH 8.0 for 16 h at 37°C. The peptides were extracted three times with 30 µl of 5% trifluoroacetic acid in 50% acetonitrile and the extract was concentrated in a speedvac. The obtained solutions were loaded onto the MALDI target plate by mixing 0.3 µl of each solution with the same volume of matrix solution (10 mg/ml α-cyanohydroxycinnaminic acid in acetonitrile/H_2_O (1∶1, v/v) and allowed to dry. Measurements were performed using a Voyager 4182 MALDI-TOF instrument (Applied Biosystems, Darmstadt, Germany), operating in the positive ion reflector mode with an accelerating voltage of 25 kV. The laser wavelength was 337 nm and the laser repetition rate was 20 Hz. The final mass spectra were produced by averaging 60 laser shots. Each spectrum was internally calibrated with the masses of two trypsin autolysis products. For peptide mass fingerprinting identification, the tryptic peptide mass maps were searched against Swiss-Prot (http://www.expasy.uniprot.org) and PlasmoDB (http://www.plasmodb.org) databases by using the search engine Protein Prospector MS-Fit. Standard search parameters were set to allow a mass accuracy of 15 ppm and two missed tryptic cleavages.

### PCR amplification, sequencing, and subcloning of *PfSAHH* and *PfOAT*

For cloning procedures of *PfSAHH*, restriction sites were introduced for *Eco*RI (underlined) and *Nco*I (italic), and for *Eco*RI (underlined) and *Xho*I (italic), respectively, at the 5′-ends of the respective primers (primer for *PfSAHH*: N-terminal: 5′-GGGCGAATTC *CCATGG*TTGAAAATAAGAGTAAGGTC-3′; C-terminal: 5′-CGCGGAATTCCTCGAGATATCTGTATTCGTTACTCT-3′). For cloning procedures of *PfOAT* the primers contained restriction sites for *Nco*I (underlined) and *Xho*I (italic) (primer for *PfOAT*: N-terminal: 5′-AGCCCATGGATTTCGTTAAAGAATTAAAAAGTAG-3′; C-terminal: 5′-ACG*CTCGAG*TAAATTGTCATCAAAAAATTTAACAG-3′). The genes for *PfSAHH* and *PfOAT* were amplified by PCR using a gametocyte cDNA library from *P. falciparum* strain 3D7 as a template. The derived fragment of *PfSAHH* of correct size was cloned into pHSG398 with *Eco*RI; the fragment of *PfOAT* was cloned into pDrive. Both fragments were sequenced and subcloned into pET28a using *Nco*I and *Xho*I.

### Heterologous expression of *PfSAHH* and *PfOAT* and purification of the proteins

*PfSAHH* and *PfOAT* were expressed in the *E. coli* strain BL 21. Cells containing the plasmid of *PfSAHH* were grown in Terrific Broth medium supplemented with kanamycin (25 µg/ml). BL 21 cells containing *PfOAT* were cultivated in Luria Bertani medium in the presence of kanamycin (25 µg/ml). The cells were grown at 23°C to an optical density at 600 nm of 0.7, and expression was subsequently induced for 15 h with 0.2 mM (*PfSAHH*) or 0.5 mM (*PfOAT*) isopropyl-β-D-1-thiogalactopyranoside. Cells were harvested, resuspended in 50 mM sodium phosphate, 300 mM NaCl, pH 8.0, and sonicated in the presence of protease inhibitors. After centrifugation, the supernatant was applied to a Ni-NTA column, and recombinant proteins were eluted with sodium phosphate buffer containing 50 mM (*PfSAHH*) or 100 mM (*PfOAT*) imidazole.

### BIAcore surface plasmon resonance detection

Surface plasmon resonance experiments were performed using a BIAcore X biosensor system (Biacore AB, Uppsala, Sweden). The carboxymethylated surface of the sensor chip CM5 was activated with a 1∶1 mixture of 0.1 M N-hydroxysuccinimide (NHS) and 0.4 M 1-ethyl-3-(3-dimethylaminopropyl)carbodiimide hydrochloride (EDC) (provided in the amine coupling kit; Biacore AB). Subsequently, 35 µl (60 µg/ml) of SAHH or OAT in 10 mM sodium acetate, pH 4.5 were injected to flow cell 2 (FC2) to be immobilized on the sensor surface via primary amine groups. No protein was injected into FC1. Residual unreacted active ester groups were blocked with 1 M ethanolamine-HCl, pH 8.5 (amine coupling kit). Experiments were performed at 25°C in HBS buffer (10 mM HEPES, 150 mM NaCl, 3.4 mM EDTA, 0.005% Nonidet P-40, pH 7.4) at a flow rate of 10 µl/min. To analyze the binding of any of the redox-active proteins (-mutants) with SAHH or OAT, the following cycles were conducted: Firstly, SAHH or OAT on the sensor surface was oxidized using 30 µl of 0.5 mM 5,5′-dithiobis(2-nitrobenzoate) (DTNB) before 30 µl of the analyte (10 µM in HBS buffer) were injected, followed by buffer flow over the chip and an elution step with 2 mM DTT in a volume of 30 µl. In order to analyze a non-covalent interaction of Grx with SAHH or OAT, 100 µM of the Grx^C29S/C32S^ mutant were treated with 10 mM iodoacetamide for 2 h in the dark. Excess iodoacetamide was eliminated by gelfiltration chromatography using NAP-5 columns (Amersham Biosciences). Difference resonance spectra (FC2–FC1) were recorded. After equilibrating the surface with HBS buffer it was ready for the next cycle. Data were evaluated using the software BIAevaluation 3.0 (Biacore AB).

### Ornithine aminotransferase assay

The conversion of ornithine to glutamate-5-semialdehyde was determined spectrophotometrically using a modified method of Kim *et al.* [65]. OAT was added to an assay mixture of 0.5 ml containing 100 mM phosphate buffer (pH 7.4), 50 mM L-ornithine, 20 mM α-ketoglutarate and 0.05 mM pyridoxal-5-phosphate and incubated for 30 min at 37°C. The reaction was stopped by adding 0.4 M HCl and 0.16% (w/v) ninhydrin. After heating for 5 min at 100°C 500 µl ethanol was added and the absorbance was measured spectrophotometrically at 512 nm.

## Supporting Information

Table S1 Oligonucleotide primers used for site-directed mutagenesis.
(0.03 MB DOC)

Figure S1 Comparison of the DTT-eluate fractions of the Trx, Grx, and Plrx pull-down experiments. The respective active site mutant of each of the three proteins was immobilized on CNBr-activated Sepharose 4B resin before incubating the column with *Plasmodium falciparum* cell lysate and washing extensively with NaCl-containing buffer. Putative target proteins were eluted with 10 mM DTT. The obtained protein samples were separated on a 12% polyacrylamide gel and stained with Coomassie blue.
(0.92 MB TIF)

Figure S2 Pull-down controls with PfTrx1 (A), PfTrx1C30S/C33S (B), PfGrx1 (C), PfGrx1C29S/C32S (D), PfPlrx (E), and CNBr-activated Sepharose 4B resin without immobilized protein (F). These control experiments were carried out in analogy to the original pull-downs; hardly any bands can be detected indicating that the binding to the single mutants of Trx, Grx, and Plrx in our pull-down experiments is specific. According to mass spectrometric analysis of the comparable band in the pull-down assays (see Figure 1), band (1) corresponds to 1-Cys peroxiredoxin, the other strong bands in the lower MW range represent the bait redoxins.
(5.68 MB TIF)
