# Supplementary material for: Identification of Proteins Targeted by the Thioredoxin Superfamily in Plasmodium falciparum
Source: PLoS Pathog. 2009 Apr 10;5(4):e1000383. doi: 10.1371/journal.ppat.1000383 (PMC2660430; doi:10.1371/journal.ppat.1000383)
Supplement: Table S1 — Oligonucleotide primers used for site-directed mutagenesis. (0.03 MB DOC) [file ppat.1000383.s001.doc]

**Supplementary Table 1.** Oligonucleotide primers used for site-directed mutagenesis

| **Mutation** | **Template** |  | **Sequence^a^** |
| --- | --- | --- | --- |
| PfTrx1^C33S^ | pQE30/*PfTrx1* | Sense | 5'-GACCAT*C*CAAAAGAATTGCCCC-3' |
|  |  | Antisense | 5'-GGGGCAATTCTTTTG*G*ATGGTC-3' |
| PfTrx1^C30S/C33S^ | pQE30/ *PfTrx1^C33S^* | Sense | 5'-CTGAATGG*A*GTGGACCAAGCAAAAG-3' |
|  |  | Antisense | 5'-CTTTTGCTTGGTCCAC*T*CCATTCAG-3' |
| PfGrx1^C32S^ | pQE30/*PfGrx1* | Sense | 5'-CGGAATGCCCATAT*A*GTATTAAGGC-3' |
|  |  | Antisense | 5'-GCCTTAATAC*T*ATATGGGCATTCCG-3' |
| PfGrx1^C29S/C32S^ | pQE30/*PfGrx1^C32S^* | Sense | 5'-GCAAAAACGGAA*A*GCCCATATAGTATT-3' |
|  |  | Antisense | 5'-AATACTATATGGGC*T*TTCCGTTTTTGC-3' |
| Plrx^C63S^ | pQE30/*Plrx* | Sense | 5'-GGTGTAAATAC*A*GTGTAACCTTTATAG-3' |
|  |  | Antisense | 5'-CTATAAAGGTTACAC*T*GTATTTACACC-3' |

^a^ Codons with mutated base pairs are underlined; the mutated base is highlighted in italics.
