# Supplementary figures and images for: Identification of Proteins Targeted by the Thioredoxin Superfamily in Plasmodium falciparum
Source: PLoS Pathog. 2009 Apr 10;5(4):e1000383. doi: 10.1371/journal.ppat.1000383 (PMC2660430; doi:10.1371/journal.ppat.1000383)

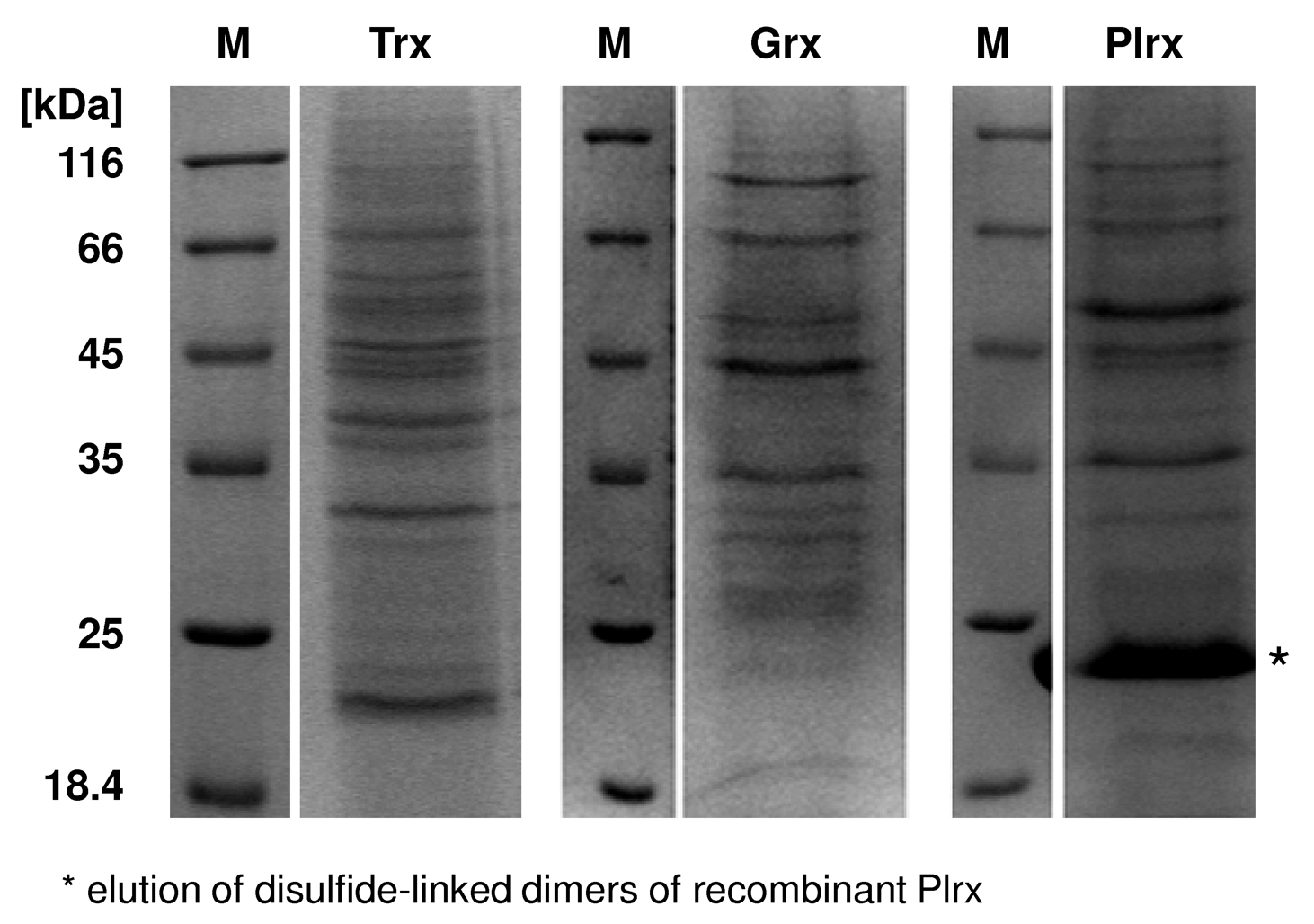

Supplement: Figure S1 — Comparison of the DTT-eluate fractions of the Trx, Grx, and Plrx pull-down experiments. The respective active site mutant of each of the three proteins was immobilized on CNBr-activated Sepharose 4B resin before incubating the column with Plasmodium falciparum cell lysate and washing extensively with NaCl-containing buffer. Putative target proteins were eluted with 10 mM DTT. The obtained protein samples were separated on a 12% polyacrylamide gel and stained with Coomassie blue. (0.92 MB TIF) [file ppat.1000383.s002.tif]
